# Social Determinants of Health and Geographic Variation in Medicare per Beneficiary Spending

**DOI:** 10.1001/jamanetworkopen.2021.13212

**Published:** 2021-06-10

**Authors:** Yongkang Zhang, Jing Li, Jiani Yu, Robert Tyler Braun, Lawrence P. Casalino

**Affiliations:** 1 Division of Health Policy and Economics, Department of Population Health Sciences, Weill Cornell Medical College, New York, New York

## Abstract

**Importance:**

Despite substantial geographic variation in Medicare per beneficiary spending in the US, little is known about the extent to which social determinants of health (SDoH) are associated with this variation.

**Objective:**

To determine the associations between SDoH and county-level price-adjusted Medicare per beneficiary spending.

**Design, Setting, and Participants:**

This cross-sectional study used county-level data on 2017 Medicare fee-for-service (FFS) spending, patient demographic characteristics (eg, age and gender) and clinical risk score, supply of health care resources (eg, number of hospital beds), and SDoH measures (eg, median income and unemployment rate) from multiple sources. Multivariable regressions were used to estimate the association of the variation in spending across quintiles with SDoH.

**Main Outcomes and Measures:**

2017 county-level price-adjusted Medicare Parts A and B spending per beneficiary. SDoH measures included socioeconomic position, race/ethnicity, social relationships, and residential and community context.

**Results:**

Among 3038 counties with 33 495 776 Medicare FFS beneficiaries (18 352 336 [54.8%] women; mean [SD] age, 72 [1.5] years), mean Medicare price-adjusted per beneficiary spending for counties in the highest spending quintile was $3785 (95% CI, $3706-$3862) higher, or 49% higher, than spending for bottom-quintile counties (mean [SD] spending per beneficiary, $11 464 [735] vs $7679 [522]; *P* < .001). The total contribution (including through both direct and indirect pathways) of SDoH was 37.7% ($1428 of $3785) of this variation, compared with 59.8% ($2265 of $3785) by patient clinical risk, 14.5% ($549 of $3785) by supply of health care resources, and 19.8% ($751 of $3785) by patient demographic characteristics. When all factors were included within the same model, the direct contribution of SDoH was associated with 5.8% of the variation, compared with 4.6% by supply, 4.7% by patient demographic characteristics, and 62.0% by patient clinical risk.

**Conclusions and Relevance:**

These findings suggest social determinants of health are associated with considerable proportions of geographic variation in Medicare spending. Policies addressing SDoH for disadvantaged patients in certain regions have the potential to contain health care spending and improve the value of health care; patient SDoH may need to be accounted for in publicly reported physician performance, and in value-based purchasing incentive programs for health care professionals.

## Key Points

**Question:**

How much variation in Medicare per beneficiary spending across counties was associated with social determinants of health (SDoH)?

**Findings:**

In this cross-sectional study, SDoH were associated with 37.7% of variation in price-adjusted Medicare per beneficiary spending between counties in the highest and lowest quintiles of spending in 2017, including both direct contributions and indirect contributions through other factors. SDoH’s direct contribution accounted for 5.8% of the variation after controlling for patient demographic characteristics, clinical risk, and supply of health care resources.

**Meaning:**

These findings suggest that addressing SDoH is important for reducing geographic spending variation and improving the value of health care.

## Teaser

This cross-sectional study examines the association of social determinants of health with variation in Medicare per beneficiary spending at the county level.

## Introduction

Medicare spending per beneficiary varies substantially across geographic regions in the US. Since differences in patient clinical risk and demographic characteristics do not fully explain regional variation in spending, and higher spending regions did not have better health outcomes,^1,2,3,4^ this substantial variation has largely been attributed to wasteful utilization.^2,5,6,7,8^ Prominent research in the 1990s suggested that the primary driver of this spending variation was physician-induced demand reflected by the supply of local health resources, in particular hospital beds and specialist physicians.^1,9^ Despite the payment and delivery reforms in the past decade, geographic variation in Medicare spending only had limited reduction.^10^

Social determinants of health (SDoH) are important drivers of health care utilization and spending.^11,12^ The association between SDoH and geographic variation in Medicare spending is important but largely unexplored. First, if SDoH are associated with a comparable or higher share of variation in Medicare spending relative to supply factors, the current payment and delivery reforms may have limited impact on reducing unnecessary spending. Second, a better understanding of SDoH’s contribution to patients’ clinical risk and health care spending could improve risk adjustment or peer group comparisons in value-based purchasing programs, avoiding penalizing health professionals who treat a large proportion of socially disadvantaged patients. Third, communities with disadvantaged social conditions (eg, higher rates of poverty and unemployment) are likely to incur higher spending because of either a higher prevalence of chronic conditions or SDoH-related barriers to care access and treatment adherence. Distinguishing between the 2 mechanisms is important for informing optimal policy and clinical interventions to address health disparities.

Using county-level Medicare spending and SDoH data from 2017, we examined the extent to which geographic variation in Medicare per beneficiary total spending across counties was associated with SDoH relative to other patient characteristics and to the supply of health care resources. We used a comprehensive group of SDoH measures and distinguished between the direct and indirect pathways (such as that mediated through clinical risk) in which SDoH may be associated with health care spending.

## Methods

### Study Data and Population

We used Medicare spending and patient demographic data of Medicare fee-for-service (FFS) patients from the Centers for Medicare & Medicaid Services (CMS) geographic variation public use file for 2017.^13^ These data include patients continuously enrolled in Medicare FFS Parts A and B in 2017. We obtained data on the supply of health care resources in 2017 from the Area Health Resource Files^14^ and the Medicare Provider of Services files.^15^ SDoH data came from the American Community Survey^16^ and the Robert Wood Johnson Foundation County Health Rankings.^17^ Our unit of analysis was at the county level since it is the most granular level at which the SDoH, supply of health care, and other variables in our analyses are available, and it is also the level at which many government policies and interventions are set to address vulnerable SDoH.^18,19^

We obtained data for all 3143 counties in the 50 US states and District of Columbia. After excluding counties with missing values from any of the data sources and counties with fewer than 100 FFS beneficiaries, our final analysis included 3038 counties. We followed the Strengthening the Reporting of Observational Studies in Epidemiology (STROBE) reporting guideline for cross-sectional studies. Our study was deemed exempt from review by the Weill Cornell Medical College institutional review board as we used publicly available data only.

### Study Variables

#### Medicare per Beneficiary Spending

Our primary spending outcome was price-adjusted Medicare total spending per FFS beneficiary in each county.^20^ This measure was published by CMS and includes spending for all Medicare Parts A and B services, accounting for differences in Medicare payment rates across regions (eg, differences in local wages).

#### Social Determinants of Health

We used a 4-step approach to select SDoH measures. First, we identified 87 SDoH measures that are publicly available at the county level through web searches and literature reviews. Second, we adapted a conceptual framework developed by the National Academy of Medicine, which specifies 5 categories of SDoH associated with Medicare spending, including socioeconomic position, race and ethnicity composition, social relationships, overall residential and community context, and gender.^21^ Third, we mapped one or more county-level SDoH measures identified in step 1 to each of the first 4 categories (gender was included in patient demographic characteristics). Fourth, we tested the correlation between SDoH measures within each category. For each group of measures that captured similar concepts and were highly correlated (ie, correlation coefficient over 0.7),^22^ we selected 1 that was most commonly used in the literature. Details about the SDoH selection process are available in the eAppendix 1 in the Supplement.

This approach yielded 13 SDoH measures at the county level: 5 for socioeconomic position (median household income, percentage of residents who are uninsured, unemployment rate, percentage of residents without a high school degree, and food environment index); 4 for race/ethnicity (percentage of residents who are Hispanic, percentage of residents who are non-Hispanic Black, percentage of residents who are non-Hispanic and of another race category [ie, American Indian and Alaska Native, Asian, Native Hawaiian and Other Pacific Islander, or other], and percentage of residents who are noncitizen); 1 for social relationships (number of membership associations per 1000 population); and 3 for residential and community context (percentage of households with severe housing problems, percentage of residents with access to exercise opportunities, and percentage of housing units in rural areas). Details about the source of each SDoH measure are available in the appendix (eTable 6 in the Supplement).

#### Patient Demographics

County-level patient demographic characteristics included a third order polynomial of mean age (to account for the nonlinear association between age and Medicare spending) and percentage of women among Medicare FFS beneficiaries in a county. We did not include share of Medicare FFS population under age 65 years because the geographic variation of this share is largely determined by socioeconomic factors.^23^

#### Clinical Risk

We used county-level mean CMS hierarchical condition category (HCC) score across all Medicare beneficiaries within a county as a measure of patient clinical risk.^24^ The CMS-HCC score is calculated based on individual demographic characteristics (age, gender, and their interactions) and the *International Statistical Classification of Diseases and Related Health Problems, Tenth Revision* (*ICD-10*) diagnosis codes in the prior year. A higher CMS-HCC score indicates that beneficiaries are less healthy and have higher anticipated health care costs.

#### Supply of Health Care Resources

Consistent with previous literature,^2,5,6,25^ we used the following county-level health care resource supply measures per 1000 population in 2017: total primary care physicians, specialists, hospital beds, skilled nursing facility beds, home health agency aides, registered nurses employed by hospices, and ambulatory care centers. These measures reflect supply across the continuum of care.

### Statistical Analysis

#### Main Analysis

We categorized counties into quintiles based on their price-adjusted per beneficiary Medicare spending in 2017. We calculated the unadjusted geographic variation in Medicare per beneficiary spending as the difference in mean spending between counties in the top quintile (quintile 5) and the bottom quintile (quintile 1). We examined the association of geographic variation in mean spending differences with SDoH as compared with the 3 other groups of characteristics (ie, demographic characteristics, clinical risk, and supply). This measure of geographic variation in spending has been used in prior studies^5,26,27,28^ and allows us to graphically visualize changes in spending variation after accounting for SDoH and other factors.

SDoH may be associated with Medicare spending through multiple pathways.^21^ First, the association between SDoH and spending may be *indirect*; for example, SDoH are associated with chronic illnesses that raise spending, such as diabetes, heart conditions, and cancer.^29,30,31^ Adjusting for clinical risk would therefore be expected to decrease the geographic variation in spending associated with SDoH. Second, SDoH could have *direct* associations with spending, independent of other patient and supply characteristics. For example, socioeconomic disadvantage may be a direct barrier to patient adherence to medical advice, leading to lower effectiveness of treatment and higher spending.^32,33^

We examined the total variation in Medicare per beneficiary spending associated with SDoH (or total contribution of SDoH to spending variation) through direct and indirect pathways and compared this with the variation associated with patient and supply characteristics. To measure the total contribution, we estimated a linear regression model with price-adjusted per beneficiary Medicare spending as the dependent variable and the 13 SDoH measures as independent variables, without controlling for any other factors. The sum of the county-level mean Medicare per beneficiary spending and the regression residual for each county represents spending in that county after adjusting for variation in SDoH.^2^ The differences between unadjusted and adjusted spending variation across quintiles represent the total variation associated with SDoH. We repeated this process for each of the other 3 groups of variables (patient demographic, clinical risk, and supply characteristics) to obtain the total contribution of each group to geographic variation in spending (eAppendix 2 in the Supplement).

To measure the direct contribution of SDoH to variation in spending, we estimated a single linear model including SDoH and the other 3 groups of characteristics (patient demographic, clinical risk, and supply characteristics) all included as independent variables. We then replaced the values of SDoH for each county with their means across all counties (without changing the values of other covariates) and estimated the adjusted per beneficiary Medicare spending.^34^ The reduction in spending variation after this adjustment reflects the amount of variation directly associated with SDoH. We repeated this process for each of the other 3 groups of characteristics (using the same regression model) in order to isolate and compare the direct contribution of each group.

#### Sensitivity and Supplementary Analyses

CMS-HCC scores rely on diagnosis codes of comorbidities within the claims database. These scores may reflect medical treatment or diagnosis intensity across regions beyond actual patient clinical risk.^35,36^ Therefore, adjusting for CMS-HCC scores may overestimate the variation in spending associated with clinical risk. To address this issue, we conducted 2 sets of sensitivity analysis. First, we estimated an alternative model with the same outcome that did not include the CMS-HCC score. This model was then used to calculate the direct contribution to geographic variation in Medicare spending of patient demographic and supply characteristics and SDoH. Second, we used the price- and risk-adjusted per beneficiary spending measure from the Dartmouth Atlas, which only adjusts for price and patient-level demographic characteristics (age, gender, and race) instead of claims-based risk score as the dependent variable, and examined the direct contribution of supply characteristics and SDoH. Details are available in eAppendix 2 and eTable 7 in the Supplement.

We conducted a supplementary analysis by estimating the association between clinical risk and SDoH, in which the dependent variable was the county-level mean CMS-HCC score and the independent variables were SDoH measures, with and without controlling for patient demographic and supply characteristics. By directly examining this association, this analysis further informs the extent to which the association between SDoH and spending was mediated by clinical risk.

All regressions used heteroskedasticity-robust standard errors. We weighted regressions by the number of FFS beneficiaries in each county so that the results will not be overly influenced by small counties. All analysis was conducted using Stata statistical software version 16 IC (StataCorp), with 2-sided hypothesis tests and *P* < .05 considered statistically significant.

## Results

### Medicare Spending Variation and County Characteristics

Our study population included 33 495 776 Medicare FFS beneficiaries (15 143 440 [45.2%] men and 18 352 336 [54.8%] women; county-level mean [SD] age, 72 [1.5] years) residing in 3038 counties. County-level price-adjusted per beneficiary Medicare spending ranged from $4447 to $16 570, a nearly 4-fold variation (Table 1). The mean (SD) per beneficiary spending among counties in the highest-spending quintile (quintile 5) was $11 464 ($735), which was $3785 (95% CI, $3706-$3862) or 49.3% higher than average per beneficiary spending among counties in the lowest-spending quintile (quintile 1) (mean [SD] per beneficiary spending, $7679 [$522]).

**Table 1.** County Characteristics by Geographic Price-Adjusted per Beneficiary Medicare Spending Quintiles^a^

Characteristic	Mean (SD)						Difference between quintiles 5 and 1 (95% CI)	*P* value
	Total (n = 3038)	Price-adjusted per beneficiary Medicare spending quintiles						
		1 (n = 608)	2 (n = 608)	3 (n = 607)	4 (n = 608)	5 (n = 607)		
Medicare cost, $								
Price-adjusted per beneficiary spending, range	4447-16 570	4447-8308	8311-9109	9111-9799	9802-10 578	10 579-16 570	NA	NA
Price-adjusted per beneficiary spending	9784 (1328)	7679 (522)	8763 (214)	9463 (183)	10 179 (216)	11 464 (735)	3785 (3706 to 3862)	<.001
Demographic characteristics								
Age, y	71.6 (1.5)	71.6 (1.3)	71.4 (1.5)	71.4 (1.4)	71.5 (1.6)	71.8 (1.5)	0.17 (−0.01 to 0.34)	.049
Women, %	54.8 (1.8)	53.2 (1.8)	54.2 (1.8)	54.8 (1.6)	55.3 (1.7)	55.5 (1.5)	2.29 (2.10 to 2.48)	<.001
Clinical risk								
CMS-HCC score	1.0 (0.1)	0.9 (0.1)	0.9 (0.1)	1.0 (0.1)	1.0 (0.1)	1.1 (0.1)	0.22 (0.21 to 0.23)	<.001
Supply of health resources per 1000 population, No.								
PCPs	0.8 (0.3)	0.9 (0.3)	0.7 (0.4)	0.7 (0.3)	0.8 (0.3)	0.7 (0.2)	−0.18 (−0.21 to −0.15)	<.001
Specialists	2.0 (1.5)	2.0 (1.7)	1.5 (1.3)	1.8 (1.5)	2.3 (1.9)	2.0 (1.2)	0.04 (−0.12 to 0.21)	.61
Hospital beds	2.5 (2.1)	2.0 (1.9)	2.2 (2.1)	2.3 (2.3)	2.8 (1.9)	2.8 (2.1)	0.82 (0.57 to 1.06)	<.001
SNF beds	5.0 (3.0)	3.7 (2.7)	5.2 (3.2)	5.1 (3.1)	5.6 (3.0)	5.1 (2.7)	1.31 (0.99 to 1.64)	<.001
HHA aides	0.5 (1.4)	0.2 (0.3)	0.5 (2.7)	0.4 (0.7)	0.8 (1.7)	0.4 (0.6)	0.24 (0.18 to 0.30)	<.001
Hospice RNs	0.18 (0.17)	0.15 (0.13)	0.15 (0.15)	0.18 (0.16)	0.20 (0.20)	0.18 (0.17)	0.03 (0.01 to 0.05)	.003
ASCs	0.02 (0.02)	0.02 (0.01)	0.02 (0.02)	0.02 (0.02)	0.02 (0.02)	0.02 (0.01)	−0.001 (−0.003 to 0.000)	.06
Social determinants of health								
Socioeconomic position								
Median household income, $	60 357 (16 255)	66 384 (19 451)	62 419 (18 400)	61 482 (15 159)	60 716 (15 529)	55 548 (13 387)	−10 835 (−12 721 to −8949)	<.001
Uninsured rate, %	10.0 (4.6)	7.8 (3.1)	8.0 (3.3)	8.9 (3.7)	9.4 (4.0)	13.2 (5.0)	5.42 (4.88 to 5.97)	<.001
Unemployment rate, %	4.4 (1.3)	4.1 (1.4)	4.3 (1.7)	4.2 (1.0)	4.5 (1.3)	4.7 (0.9)	0.62 (0.49 to 0.75)	<.001
Without high school degree, %	12.8 (5.5)	10.1 (4.5)	11.1 (5.7)	11.3 (4.3)	12.6 (4.8)	15.9 (5.7)	5.82 (5.18 to 6.47)	<.001
Food environment index	7.84 (0.87)	8.06 (0.73)	8.13 (0.78)	7.94 (0.81)	7.79 (0.86)	7.57 (0.92)	−0.49 (−0.60 to −0.39)	<.001
Race/ethnicity, %								
Hispanic	17.7 (17)	15.1 (13.4)	11.8 (13.3)	13.4 (11.9)	16.3 (17.5)	25.8 (19.5)	10.71 (8.55 to 12.88)	<.001
Non-Hispanic Black	12.3 (12.6)	3.2 (3.3)	6.4 (7.2)	10.2 (10.8)	14.7 (14.0)	18.6 (13.1)	15.48 (14.14 to 16.81)	<.001
Non-Hispanic other race	0.2 (0.3)	0.2 (0.2)	0.2 (0.2)	0.2 (0.2)	0.2 (0.3)	0.3 (0.3)	0.13 (0.09 to 0.16)	<.001
Noncitizens, %	7.0 (5.5)	6.7 (5.1)	4.8 (4.5)	5.5 (3.8)	6.2 (4.6)	9.9 (6.4)	3.18 (2.45 to 3.92)	<.001
Social relationships								
Social associations per 1000 population	9.27 (3.89)	9.63 (3.69)	10.61 (4.17)	9.83 (3.84)	9.78 (3.92)	7.66 (3.30)	−1.98 (−2.39 to −1.56)	<.001
Residential and community context, %								
Households with severe housing problems	18.6 (6.0)	18.6 (4.8)	15.5 (4.4)	16.3 (3.9)	18.1 (5.0)	22.2 (7.1)	3.53 (2.74 to 4.32)	<.001
Access to exercise opportunities	84.2 (16.0)	85.3 (14.0)	80.9 (16.0)	82.5 (15.6)	84.3 (15.9)	86.4 (16.9)	1.06 (−0.89 to 3.00)	.29
Housing units in rural areas	18.8 (24.4)	25.8 (27.2)	29.6 (27.1)	21.1 (23.2)	16.2 (22.4)	11.2 (20.8)	−14.62 (−17.41 to −11.83)	<.001

Abbreviations: ASC, ambulatory surgery center; CMS-HCC, Centers for Medicare & Medicaid Services hierarchical condition category; HHA, home health agency; PCP, primary care physicians; NA, not applicable; RN, registered nurses; SNF, skilled nursing facility.

^a^ For Medicare cost and demographics, analyses were weighted by the number of fee-for-service beneficiaries in each county; for supply and social determinants, analyses were weighted by the population size in each county.

Compared with counties in quintile 1, patients in quintile 5 were more likely to be women (55.5% vs 53.2%) and had a higher mean (SD) CMS-HCC score (1.1 [0.09] vs 0.9 [0.07]). Counties in quintile 5 had fewer primary care physicians but more acute and postacute beds and specialists. Quintile 5 counties had lower median household income (mean [SD], $55 548 [$13 387] vs $66 384 [$19 451]), higher uninsured (13.2% vs 7.8%) and unemployment (4.7% vs 4.1%) rates, and a lower food environment index (7.6 [0.9] vs 8.1 [0.7]) compared with quintile 1 counties. In addition, quintile 5 counties also had more residents who identified as part of a racial/ethnic minority group (eg, 25.8% vs 15.1% for Hispanic ethnicity) or not US citizens (9.9% vs 6.7%), fewer membership associations (7.7 [3.3] vs 9.6 [3.7] per 1000 population), and more households with severe housing problems (22.2% vs 18.6%). All differences were statistically significant (*P* < .001).

### Explaining Geographic Variation in Medicare Spending

#### Total Contribution

**Figure 1.**
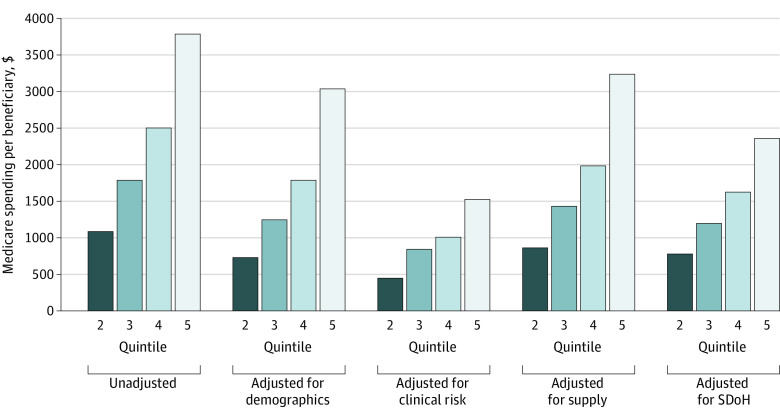
Variation in Price-Adjusted per Beneficiary Medicare Spending Between the Counties in Quintile 1 and Counties in Quintiles 2 Through 5 in 2017 SDoH indicates social determinant of health.

**Figure 2.**
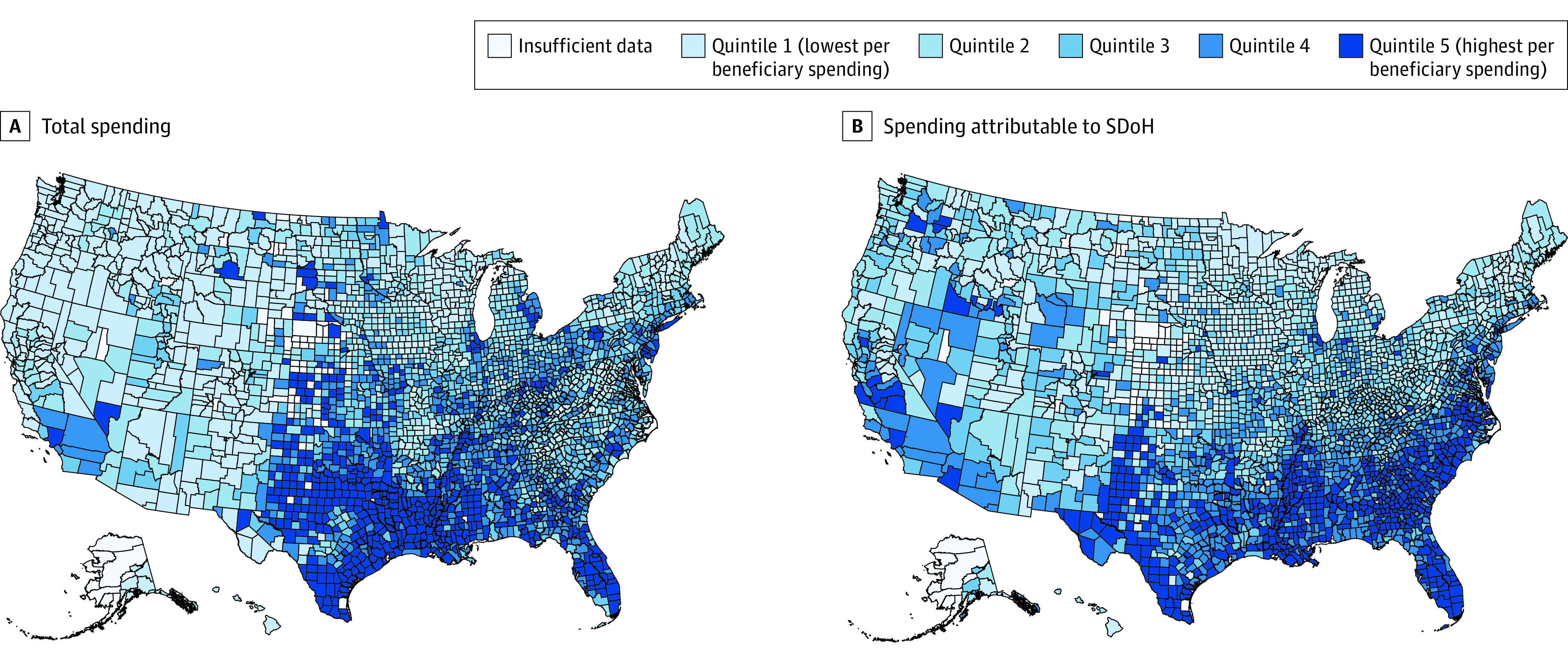
Maps of Quantiles of 2017 County-Level Price-Adjusted per Beneficiary Medicare Spending SDoH indicates social determinant of health.

Adjusting for SDoH alone reduced 37.7% of variation ($1428 of $3785) between high-spending (quintile 5) and low-spending counties (quintile 1), and 28.1% to 35.0% of variation between quintiles 2 through 4 and quintile 1 (*P* < .001) (Figure 1 and Table 2). In comparison, adjusting for clinical risk alone reduced the variation between counties in quintile 5 and those in quintile 1 by 59.8% ($2265 of $3785), and reduced the variation between quintiles 2 and 4 and quintile 1 by 52.9% to 59.8% (*P* < .001). Demographic characteristics alone and supply of health resources alone were respectively associated with 19.8% ($751 of $3785; *P* < .001) and 14.5% ($549 of $3785; *P* < .001) of the variation between quintile 5 and quintile 1 counties. Figure 2 show the quintiles of county-level per beneficiary Medicare total spending and spending attributable to SDoH.

**Table 2.** Full Regression Output of Primary Analysis^a^

Characteristics	Coefficient for adjustment (95% CI)					Partial *F*	*P* value
	Demographic characteristics (n = 3038)	Clinical risk (n = 3038)	Supply (n = 3038)	SDoH (n = 3038)	All factors (n = 3038)		
Demographic							
Age	−91 837.72 (−254 492.60 to 70 817.15)	NA	NA	NA	−76 419.42 (−137 892.80 to −14 946.03)	4.7	<.001
*P* value	.27	NA	NA	NA	.02		
Age squared	1265.28 (−1046.88 to 3577.45)	NA	NA	NA	1079.54 (204.53 to 1954.56)		
*P* value	.28	NA	NA	NA	.02		
Age cubed	−5.812 (−16.76 to 5.14)	NA	NA	NA	−5.07 (−9.22 to −0.92)		
*P* value	.30	NA	NA	NA	.02		
Women	325.20 (279.74 to 370.65)	NA	NA	NA	68.58 (28.12 to 109.04)		
*P* value	<.001	NA	NA	NA	.001		
Clinical risk							
CMS-HCC score	NA	10 479.92 (9732.73 to 11 227.11)	NA	NA	10 852.63 (9890.81 to 11 814.44)	489.5	<.001
*P* value	NA	<.001	NA	NA	<.001		
Supply of health resources per 1000 population							
PCPs	NA	NA	−2330.73 (−2793.61 to −1867.86)	NA	−493.47 (−701.08 to −285.86)	12.1	<.001
*P* value	NA	NA	<.001	NA	<.001		
Specialists	NA	NA	415.71 (292.35 to 539.07)	NA	29.28 (−10.17 to 68.74)		
*P* value	NA	NA	<.001	NA	.15		
Hospital beds	NA	NA	47.74 (18.01 to 77.46)	NA	15.32 (1.58 to 29.05)		
*P* value	NA	NA	.002	NA	.03		
SNF beds	NA	NA	38.88 (14.88 to 62.89)	NA	40.16 (27.45 to 52.87)		
*P* value	NA	NA	.002	NA	<.001		
HHA aides	NA	NA	32.16 (−17.89 to 82.21)	NA	13.28 (−9.19 to 35.75)		
*P* value	NA	NA	.21	NA	.25		
Hospice RNs	NA	NA	596.55 (213.61 to 979.49)	NA	401.13 (201.98 to 600.27)		
*P* value	NA	NA	.002	NA	<.001		
ASCs	NA	NA	4423.34 (−734.08 to 9580.77)	NA	−350.03 (−2872.56 to 2172.51)		
*P* value	NA	NA	.09	NA	.79		
**Social determinants of health**							
Socioeconomic position							
Median household income, $	NA	NA	NA	−0.0065 (−0.019 to 0.006)	0.012 (0.005 to 0.019)	23.8	<.001
*P* value	NA	NA	NA	.32	<.001		
Uninsured rate	NA	NA	NA	99.74 (72.06 to 127.43)	92.78 (77.11 to 108.45)		
*P* value	NA	NA	NA	<.001	<.001		
Unemployment rate	NA	NA	NA	10.48 (−68.78 to 89.74)	−4.21 (−51.99 to 43.58)		
*P* value	NA	NA	NA	.80	.86		
% Without high school degree	NA	NA	NA	69.43 (30.27 to 108.59)	−20.19 (−37.23 to −3.15)		
*P* value	NA	NA	NA	.001	.20		
Food environment index	NA	NA	NA	303.41 (126.71 to 480.10)	−37.84 (−132.90 to 57.22)		
*P* value	NA	NA	NA	.001	.44		
Race/ethnicity, %							
Non-citizens	NA	NA	NA	−30.71 (−80.54 to 19.11)	−27.48 (−54.12 to −0.83)		
*P* value	NA	NA	NA	.23	.04		
Hispanic	NA	NA	NA	−7.62 (−18.59 to 3.35)	3.39 (−3.94 to 10.73)		
*P* value	NA	NA	NA	.17	.36		
Non-Hispanic black	NA	NA	NA	30.69 (21.76 to 39.62)	3.41 (−1.71 to 8.53)		
*P* value	NA	NA	NA	<.001	.19		
Non-Hispanic other race	NA	NA	NA	221.12 (−96.33 to 538.57)	−59.27 (−337.67 to 219.14)		
*P* value	NA	NA	NA	.17	.68		
Social relationships							
Per 1000 population	NA	NA	NA	0.56 (−16.74 to 17.86)	−24.35 (−37.80 to −10.90)		
*P* value	NA	NA	NA	.95	<.001		
Residential and community context, %							
Households with severe housing problems	NA	NA	NA	0.91 (−48.15 to 49.98)	−34.84 (−55.16 to −14.57)		
*P* value	NA	NA	NA	.97	.001		
Residents with access to exercise opportunities	NA	NA	NA	−5.25 (−10.00 to −0.49)	−2.57 (−6.31 to 1.17)		
*P* value	NA	NA	NA	.03	.18		
Housing units in rural areas	NA	NA	NA	−20.48 (−24.25 to −16.71)	−0.47 (−3.54 to 2.61)		
*P* value	NA	NA	NA	<.001	.76		
Overall F	66.6	756.3	22.6	60.80	121.0	NA	NA
*P* value of F	<.001	<.001	<.001	<.001	<.001	NA	NA
*R^2^*	0.18	0.62	0.14	0.38	0.77	NA	NA
Adjusted *R^2^*	0.18	0.62	0.14	0.38	0.77	NA	NA

Abbreviations: ASC, ambulatory surgery center; CMS-HCC, Centers for Medicare & Medicaid Services hierarchical condition category; HHA, home health agency; NA, not applicable; PCP, primary care physicians; RN, registered nurses; SNF, skilled nursing facility.

^a^ Results are from the linear regressions using CMS price-adjusted per beneficiary Medicare spending as outcome, adjusting for variables in each column.

#### Direct Contribution

**Figure 3.**
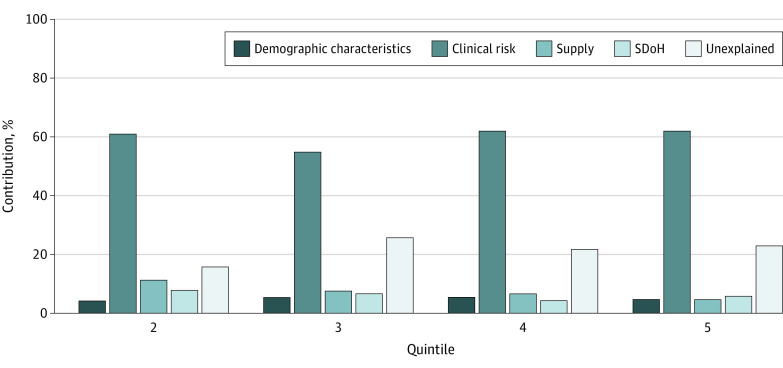
Direct Contributions to Variation in Price-Adjusted per Beneficiary Medicare Spending Between Quintiles 2 Through 5 and Quintile 1, 2017 SDoH indicates social determinant of health.

In the multivariable regression including all 4 groups of characteristics as independent variables, the direct contribution of SDoH to variation in spending between counties in quintile 5 and those in quintile 1 was 5.8% ($219 of $3785; *P* < .001) compared with 4.6% for supply characteristics ($175 of $3785; *P* < .001) (Figure 3). The direct contribution of demographic characteristics was 4.7% ($179 of $3785; *P* < .001). Clinical risk had the highest direct contribution to variation in price-adjusted per beneficiary spending, associated with 62.0% of the variation between counties in quintile 5 and those in quintile 1 ($2345 of $3785; *P* < .001). The relative magnitude of direct contributions across groups of factors was similar for spending variation between counties in quintiles 2 through 4 and quintile 1. Approximately 22.9% of variation between quintile 5 and quintile 1 remained after adjusting for all factors.

### Sensitivity and Supplementary Analyses

The direct contributions of SDoH and other characteristics were sensitive to the inclusion of clinical risk score in the model. In our first sensitivity analysis excluding the CMS-HCC score from the regression model, the direct contribution of SDoH to per beneficiary spending variation between quintile 5 and quintile 1 counties rose from 5.8% to 28.2% (eFigure 1 and eTable 8 in the Supplement). The direct contributions of patient demographic and supply characteristics increased to 16.4% and 10.2%, respectively. Similarly, in our second sensitivity analysis using Dartmouth per beneficiary spending adjusted for price, age, gender, and race as the outcome, the direct contribution of SDoH to per beneficiary spending variation between quintile 5 and quintile 1 counties increased to 22.6% (eFigure 2 and eTable 8 in the Supplement).

SDoH were associated with about half of all variation in CMS-HCC score as measured by adjusted *R^2^* (eTable 9 and eFigure 3 in the Supplement). These associations remained after controlling for patient demographic and supply characteristics.

## Discussion

Our results suggest that SDoH were directly associated with 5.8% of geographic variation in Medicare spending between top- and bottom-spending quintile counties in 2017. This number increased to 37.7% when including the indirect associations between SDoH and Medicare spending through clinical risk and supply of health care resources. SDoH were associated with a similar or higher share of variation in Medicare spending compared with the supply of health care resources.

To our knowledge, this is the first study examining the associations between a comprehensive set of SDoH measures and county-level per beneficiary Medicare total spending. Our results were consistent with, but distinct from, 3 prior studies using a small number of SDoH measures and different populations and methods. Keating et al^37^ found that individual-level sociodemographic characteristics, such as marital status and income, explained a small (7%) proportion of end-of-life spending variation across hospital referral regions among 1132 cancer patients, without controlling for other factors. Sheiner^38^ found that controlling for state-level socioeconomic factors, including percentage uninsured and percentage of Black beneficiaries, greatly reduced variation in Medicare per beneficiary acute care spending across states. Zuckerman^26^ found that individual income explained less than 1% of geographic variation in Medicare per beneficiary spending.

In comparison, our study examined spending variation in smaller geographic units (ie, the county level), and used a much more comprehensive set of SDoH measures that also took into account patient demographic, clinical, and health care supply characteristics. Our study is also the first to quantify the contribution of SDoH to geographic variation in spending via direct and indirect pathways. Compared with findings from previous studies, the much higher total contribution of SDoH to geographic variation in spending identified in this study highlights the importance of measuring multiple dimensions of contextual social conditions that are associated with health care spending.

Our results suggest that failure to address SDoH may indicate missed opportunities for reducing geographic variation in spending and for reducing health disparities in regions with disadvantaged social conditions. Greater investments in job training, healthy food access, and neighborhood exercise infrastructure are all potential ways to improve SDoH, and may lead to downstream reduction of health care spending, an important consideration when evaluating the fiscal impact of these interventions. Addressing SDoH may be especially important during the COVID-19 pandemic, which disproportionately affects individuals and areas of disadvantaged social conditions.^39,40^ As a recent example, North Carolina provided financial relief to particular counties to help with rent, mortgage, medical care, or other living expenses to support successful quarantine protections during the pandemic.^18^

It is important to note that results from our county-level analyses cannot be directly extended to the individual level (the so-called “ecological fallacy”), although a prior study found that regions with high Medicare per beneficiary spending experience higher individual expenditures for all patients.^41^ The advantages of using aggregated county-level data include reduced measurement error and confounding as compared with individual-level data.^41^ In addition, identifying SDoH associated with variation in county-level Medicare per beneficiary spending is of great policy importance since government policies and interventions are often set at the county level.^18,19,42^

We found that much of the contribution of SDoH to spending variation was mediated through patient’s clinical risk, suggesting that addressing social risk factors may be important to mitigate clinical risk. Our results contribute to the debate of incorporating SDoH into Medicare value-based payment programs, such as the Merit-based Incentive Payment System. Critics suggest that disparities of care would widen if SDoH were included in the risk adjustment models of value-based payment programs, because it implies that differences in outcomes by SDoH are expected and accepted.^43^ Proponents state that risk adjustment for SDoH is essential to making fair quality performance comparisons among health care professionals treating varying proportions of patients with low socioeconomic status.^43^ Our results suggest that SDoH contribute to Medicare spending even after accounting for clinical risk and other patient characteristics, and should be accounted for in value-based payment and public reporting of health care network performance. This could be done using risk adjustment, with the pros and cons described in this paragraph, or it could be done using peer grouping (eg, comparing health care professionals with others whose patients have a similar SDoH profile, as recommended by MedPAC among others).^44^

### Limitations

This study has important limitations. First, it is associational and does not establish a causal relationship between SDoH and Medicare spending. Second, using county as the geographic unit of analysis, despite being more granular than higher levels of geographic units such as states or hospital referral regions, may overlook important variation within counties. Third, we were not able to measure certain important aspects of SDoH, such as transportation and air quality, which are associated with health care spending.^45,46^ We may have therefore underestimated the contribution of SDoH. Fourth, patient clinical risk may be a consequence of preceding SDoH exposures, and the cross-sectional nature of the study does not allow us to capture such longitudinal effects of SDoH on subsequent clinical risk and Medicare spending, which again may lead to underestimation of the contribution of SDoH. Fifth, our study focused on Medicare Parts A and B spending among Medicare FFS patients, and did not include spending on Medicare Part D or Medicare Advantage patients or other patient populations because of data limitations. Finally, we examined the contribution of multiple SDoH measures as a group to Medicare spending variation without decomposing the contribution of each individual SDoH measure, which is important although beyond the scope of this study.

## Conclusions

Our study highlights the importance of SDoH to geographic variation in Medicare spending. Addressing SDoH can provide another means to lower health care spending and presumably reduce disparities in health. Further studies are warranted to examine the impact of interventions addressing SDoH on health care spending and to test appropriate ways to incorporate social factors into risk adjustment formulas.

## Notes

Supplement. **eAppendix 1.** Selection of SDoH Variables **eTable 1.** List of Social Determinants of Health Variables Considered in the Study **eTable 2.** Correlations Between Social Determinants of Health Measures of Socioeconomic Position **eTable 3.** Correlations Between Social Determinants of Health Measures of Race and Ethnicity Composition **eTable 4.** Correlations Between Social Determinants of Health Measures of Overall Residential and Community Context **eTable 5.** Correlations Between Social Determinants of Health Measures Included in the Study **eTable 6.** Sources of Social Determinants of Health Measures Used in this Study **eAppendix 2.** Regression Models to Examine the Contribution of Patient, Supply, and SDoH Characteristics to Geographic Variation in Per Capita Medicare Total Spending **eTable 7.** Summary of Regression Models and Their Purposes **eFigure 1.** Contribution to Variation in Price-Adjusted Per Capita Medicare Spending Between Quintiles 2-5 and Quintile 1, Excluding Clinical Risk Score **eFigure 2.** Contribution to Variation in Price-, Age-, Gender-, and Race-Adjusted Per Capita Spending Between Quintiles 2-5 and Quintile 1, Using Dartmouth Spending as Outcomes **eTable 8.** Full Regression Output of Sensitivity Analysis: Coefficients and Robust Standard Errors **eTable 9.** Full Regression Output of the Association of CMS-HCC Score With SDoH, Demographics, and Supply of Healthcare Sources: Coefficients and Robust Standard Errors **eFigure 3.** Contribution to variation in CMS HCC score between quintiles 2-5 and quintile 1 eReferences.
