# Supplementary material for: Social Determinants of Health and Geographic Variation in Medicare per Beneficiary Spending
Source: JAMA Netw Open. 2021 Jun 10;4(6):e2113212. doi: 10.1001/jamanetworkopen.2021.13212 (PMC8193453; doi:10.1001/jamanetworkopen.2021.13212)
Supplement: Supplement. — eAppendix 1. Selection of SDoH Variables eTable 1. List of Social Determinants of Health Variables Considered in the Study eTable 2. Correlations Between Social Determinants of Health Measures of Socioeconomic Position eTable 3. Correlations Between Social Determinants of Health Measures of Race and Ethnicity Composition eTable 4. Correlations Between Social Determinants of Health Measures of Overall Residential and Community Context eTable 5. Correlations Between Social Determinants of Health Measures Included in the Study eTable 6. Sources of Social Determinants of Health Measures Used in this Study eAppendix 2. Regression Models to Examine the Contribution of Patient, Supply, and SDoH Characteristics to Geographic Variation in Per Capita Medicare Total Spending eTable 7. Summary of Regression Models and Their Purposes eFigure 1. Contribution to Variation in Price-Adjusted Per Capita Medicare Spending Between Quintiles 2-5 and Quintile 1, Excluding Clinical Risk Score eFigure 2. Contribution to Variation in Price-, Age-, Gender-, and Race-Adjusted Per Capita Spending Between Quintiles 2-5 and Quintile 1, Using Dartmouth Spending as Outcomes eTable 8. Full Regression Output of Sensitivity Analysis: Coefficients and Robust Standard Errors eTable 9. Full Regression Output of the Association of CMS-HCC Score With SDoH, Demographics, and Supply of Healthcare Sources: Coefficients and Robust Standard Errors eFigure 3. Contribution to variation in CMS HCC score between quintiles 2-5 and quintile 1 eReferences. [file jamanetwopen-e2113212-s001.pdf]

Supplemental Online Content

Zhang Y, Li J, Yu J, Braun RT, Casalino LP. Social determinants of health and geographic variation in Medicare per beneficiary spending. *JAMA Netw Open.* 2021;4(6):e2113212. doi:10.1001/jamanetworkopen.2021.13212

eAppendix 1. Selection of SDoH Variables

eTable 1. List of Social Determinants of Health Variables Considered in the Study

eTable 2. Correlations Between Social Determinants of Health Measures of Socioeconomic Position

eTable 3. Correlations Between Social Determinants of Health Measures of Race and Ethnicity Composition

eTable 4. Correlations Between Social Determinants of Health Measures of Overall Residential and Community Context

eTable 5. Correlations Between Social Determinants of Health Measures Included in the Study

eTable 6. Sources of Social Determinants of Health Measures Used in this Study

eAppendix 2. Regression Models to Examine the Contribution of Patient, Supply, and SDoH Characteristics to Geographic Variation in Per Capita Medicare Total Spending

eTable 7. Summary of Regression Models and Their Purposes

eFigure 1. Contribution to Variation in Price-Adjusted Per Capita Medicare Spending Between Quintiles 2-5 and Quintile 1, Excluding Clinical Risk Score

eFigure 2. Contribution to Variation in Price-, Age-, Gender-, and Race-Adjusted Per Capita Spending Between Quintiles 2-5 and Quintile 1, Using Dartmouth Spending as Outcomes

eTable 8. Full Regression Output of Sensitivity Analysis: Coefficients and Robust Standard Errors

eTable 9. Full Regression Output of the Association of CMS-HCC Score With SDoH, Demographics, and Supply of Healthcare Sources: Coefficients and Robust Standard Errors

eFigure 3. Contribution to variation in CMS-HCC score between quintiles 2-5 and quintile 1

© 2021 Zhang Y et al. *JAMA Network Open.*

This supplemental material has been provided by the authors to give readers additional information about their work.

© 2021 Zhang Y et al. *JAMA Network Open.*

eAppendix 1. Selection of SDoH Variables

We used the National Academy of Medicine (NAM) conceptual framework as the basis for selecting SDoH measures.[1] The NAM conceptual framework specifies five categories of SDoH associated with Medicare spending, including (1) *socioeconomic position*, (2) *race and ethnicity composition*, (3) *social relationships*, (4) *overall residential and community context*, and (5) *gender*. We identified 87 publicly available county-level SDoH measures from literature and web searches (eTable 1). SDoH measures identified in our searches, but not publicly available were excluded from this study (e.g., walking score, transit score, self-reported financial burden, and self-reported financial barriers to medication). After we identified publicly available SDoH measures, we mapped each of the 87 SDoH measures to one of the four NAM's conceptual framework categories and the subcategories therein (e.g. income, insurance etc. under socioeconomic position). We made two changes to the conceptual framework after this step. First, we did not use gender as one of the SDoH measures as we considered gender as part of demographics. Second, NAM's conceptual framework considered healthcare resources to be part of the *Residential and Community Context* category; however, we used healthcare resources separately to be consistent with previous literature,[2-5] which emphasized the importance of the supply of healthcare resources to regional spending variation.

After mapping of SDoH measures to the NAM conceptual framework, we qualitatively screened SDoH measures that were conceptually similar under the same category or across subcategories. For example, % receiving public assistance income, % receiving supplement security income, and % receiving food stamp/snap in the "Income" subcategory all capture poverty. We therefore only included % residents in poverty (based on federal poverty threshold). We selected up to two SDoH variables for each conceptually similar measure for further consideration.

The qualitative screening generated a total of 13 SDoH measures, generally with one measure in each subcategory (except for marital status and living alone which we did not include in any measure as they conceptually overlap with social relationships), including six for socioeconomic position (median household income, % of residents in poverty, % of residents who are uninsured, unemployment rate, % of residents without a high school degree, and food environment index), three for race and ethnicity composition (% of residents who are non-white, % of residents who are non-citizen, and % residents with limited English proficiency), one for social relationships (number of membership associations per 1,000 population), and three for overall residential and community context (% of households with severe housing problems, % of residents with access to exercise opportunities, and % of housing units in rural areas). Finally, we tested the correlation between SDoH measures within each category (eTables 2-4). For each group of measures that captured similar concepts and were highly correlated (i.e., correlation coefficient over 0.7),[6] we selected the variable that was most commonly used in the literature. Therefore, we dropped % of residents in poverty given its high correlation with median household income (eTable 2). We also dropped % residents with limited English proficiency as it

is highly correlated with % of residents who are non-citizen (eTable 3). We tested the correlation between the remaining 11 SDoH measures and included them in the analysis (eTable 5). We subsequently adopted a more detailed race/ethnicity classification and replaced the % resident who are non-White with % Hispanic, % non-Hispanic Black and % non-Hispanic with another race. Therefore, our final analyses include 13 SDoH measures.

© 2021 Zhang Y et al. *JAMA Network Open.*

eTable 1 List of social determinants of health variables considered in the study

ID	Categories and Subcategories	Measures	Sources
	**Socioeconomic Position**		
1.	Income	Median Household Income	ACS 2017 5-year Estimates
2.		% in poverty	ACS 2017 5-year Estimates
3.		% receiving public assistance income	ACS 2017 5-year Estimates
4.		% receiving supplement security income	ACS 2017 5-year Estimates
5.		% receiving food stamp/snap	ACS 2017 5-year Estimates
6.	Insurance	% Uninsured	ACS 2017 5-year Estimates
7.		% Uninsured, <18	ACS 2017 5-year Estimates
8.		% Uninsured, 18-64	ACS 2017 5-year Estimates
9.		% Uninsured, over 65	ACS 2017 5-year Estimates
10.		% Any private insurance, all ages	ACS 2017 5-year Estimates
11.		% Any public insurance, all ages	ACS 2017 5-year Estimates
12.		% Insured	ACS 2017 5-year Estimates
13.		% Medicare	ACS 2017 5-year Estimates
14.		% Medicare only	ACS 2017 5-year Estimates
15.		% Medicaid	ACS 2017 5-year Estimates
16.		% Medicaid only	ACS 2017 5-year Estimates
17.		% Medicaid, over 65	ACS 2017 5-year Estimates
18.	Education	% No schooling	ACS 2017 5-year Estimates
19.		% Completed high school, no degree	ACS 2017 5-year Estimates
20.		% High school or GED degree	ACS 2017 5-year Estimates
21.		% Some college, no degree	ACS 2017 5-year Estimates
22.		% College Degree	ACS 2017 5-year Estimates
23.		% Masters, professional, doctorate	ACS 2017 5-year Estimates
24.		% Other level of schooling (< High school)	ACS 2017 5-year Estimates
25.	Occupation	Unemployment rate	BLS, 2017 / ACS 2017 5-year Estimates
26.		White collar occupation*	ACS 2017 5-year Estimates
27.	Food	Food insecurity	County Health Ranking, 2019/Map the Meal Gap
28.		Food environment	County Health Ranking, 2019/USDA Food Environment Atlas
	**Race, Ethnicity, and Community Context**		
29.	Race and Ethnicity	% White	ACS 2017 5-year Estimates
30.		% African American	ACS 2017 5-year Estimates
31.		% American Indian	ACS 2017 5-year Estimates
32.		% Asian	ACS 2017 5-year Estimates

© 2021 Zhang Y et al. *JAMA Network Open.*

33.		% Native Hawaiian/Pacific Islander	ACS 2017 5-year Estimates
34.		% Other Race	ACS 2017 5-year Estimates
35.		% 2 or more Races	ACS 2017 5-year Estimates
36.		% Latino/Hispanic Ethnicity	ACS 2017 5-year Estimates
37.	Language	% Limited English proficiency	ACS 2017 5-year Estimates
38.		% Language other than English	ACS 2017 5-year Estimates
39.	Nativity	% Non-citizen	ACS 2017 5-year Estimates
40.		% Foreign born	ACS 2017 5-year Estimates
41.		% US citizen	ACS 2017 5-year Estimates
42.		% Native born in US	ACS 2017 5-year Estimates
43.	**Gender**	% Male	ACS 2017 5-year Estimates
44.		% Female	ACS 2017 5-year Estimates
	**Social Relationships**		
45.	Marital Status	% Now married	ACS 2017 5-year Estimates
46.		% Widowed	ACS 2017 5-year Estimates
47.		% Divorced	ACS 2017 5-year Estimates
48.		% Separated	ACS 2017 5-year Estimates
49.		% Never married	ACS 2017 5-year Estimates
50.	Living Alone	% Lives alone	ACS 2017 5-year Estimates
51.		% Householder living with spouse or spouse of householder	ACS 2017 5-year Estimates
52.		% Householder living with unmarried partner or unmarried partner of householders	ACS 2017 5-year Estimates
53.		% Child of householder	ACS 2017 5-year Estimates
54.		% Other relatives	ACS 2017 5-year Estimates
55.		% Other nonrelatives	ACS 2017 5-year Estimates
56.	Social Support	Social associations	County Health Ranking, 2019/County Business Patterns
57.		Children in single-parent households	ACS 2017 5-year Estimates
	**Residential and Community Context**		
	Built environment		
58.	Housing	Median home value	ACS 2017 5-year Estimates
59.		Median gross rent	ACS 2017 5-year Estimates
60.		Median monthly mortgage	ACS 2017 5-year Estimates
61.		Percentage of owner-occupied housing units (home ownership rate)	ACS 2017 5-year Estimates
62.		Percentage of occupied housing units without a motor vehicle	ACS 2017 5-year Estimates
63.		Percentage of occupied housing units without a telephone	ACS 2017 5-year Estimates
64.		Percentage of occupied housing units without complete plumbing	ACS 2017 5-year Estimates

© 2021 Zhang Y et al. *JAMA Network Open.*

65.		Percentage of occupied housing units with >1 person per room (crowding)	ACS 2017 5-year Estimates
66.		Total vacant addresses	USPS
67.		% of households with severe housing problems	County Health Ranking, 2019/ Comprehensive Housing Affordability Strategy (CHAS) data
68.		Low-vacancy areas	HUD, data collected annually through 2019
69.		Monthly housing costs as a percentage of household income in the past 12 months	ACS 2017 5-year Estimates
70.	Health care resources	Number of total physicians (MD+DO) per 1,000 population	Area Health Resource File
71.		Number of primary care physician per 1,000 population	Area Health Resource File
72.		Number of hospital beds per 1,000 population	Area Health Resource File
73.		Number of SNF beds per 1,000 population	Area Health Resource File
74.		Number of HHA per 1,000 population	Area Health Resource File
75.		Number of hospices per 1,000 population	Area Health Resource File
76.		Number of ambulatory surgery center per 1,000 population	Area Health Resource File
77.	Social environment	GINI inequality index	ACS 2017 5-year Estimates
78.		Urban/Rural	ACS 2017 5-year Estimates
79.		Violent crime	County Health Ranking, 2019/Uniform Crime Reporting – FBI
80.		Injury deaths	County Health Ranking, 2019
81.		Access to exercise opportunities	County Health Ranking, 2019
	**Composite Indices**		
82.	Social Vulnerability Index (SVI)	SVI-total themes percentile	Centers for Disease Control and Prevention
83.		SVI-Socioeconomic Status percentile	Centers for Disease Control and Prevention
84.		SVI-Household Composition & Disability percentile	Centers for Disease Control and Prevention
85.		SVI-Minority Status & Language percentile	Centers for Disease Control and Prevention
86.		SVI-Housing Type & Transportation percentile	Centers for Disease Control and Prevention
87.	Social Deprivation Index	Social Deprivation Index	The Robert Graham Center

Notes: ACS: American Community Survey; BLS: Bureau of Labor Statistics; USDA: United States Department of Agriculture; USPS: United States Postal Service; HUD: United States Department of Housing and Urban Development. SNF: skilled nursing facility; HHA: home health agency. * Definition from https://seer.cancer.gov/seerstat/variables/countyattribs/static.html#14-18

© 2021 Zhang Y et al. *JAMA Network Open.*

eTable 2 Correlations between social determinants of health measures of socioeconomic position

	Median household income	% of residents in poverty	% of residents who are uninsured	Unemployment rate	% of residents without a high school degree	Food environment index
Median household income	1.0000					
% of residents in poverty	**-0.7600**	1.0000				
% of residents who are uninsured	-0.3427	0.3581	1.0000			
Unemployment rate	-0.4422	0.5567	0.0907	1.0000		
% of residents without a high school degree	-0.5558	0.6396	0.5691	0.4332	1.0000	
Food environment index	0.5811	-0.6678	-0.3734	-0.4135	-0.3536	1.0000

eTable 3 Correlations between social determinants of health measures of race and ethnicity composition

	% of residents who are non-white	% of residents who are non-citizen	% residents with limited English proficiency
% of residents who are non-white	1.0000		
% of residents who are non-citizen	0.1968	1.0000	
% residents with limited English proficiency	0.1496	**0.8199**	1.0000

© 2021 Zhang Y et al. *JAMA Network Open.*

eTable 4 Correlations between social determinants of health measures of overall residential and community context

	% of households with severe housing problems	% of residents with access to exercise opportunities	% of housing units in rural areas
% of households with severe housing problems	1.0000		
% of residents with access to exercise opportunities	0.2209	1.0000	
% of housing units in rural areas	-0.3595	-0.6094	1.0000

© 2021 Zhang Y et al. *JAMA Network Open.*

eTable 5 Correlations between Social determinants of health measures included in the study

	Median household income	% of residents who are uninsured	Unemployment rate	% of residents without a high school degree	Food environment index	% of residents who are non-white	% of residents who are non-citizen	Number of membership associations per 1,000 population	% of households with severe housing problems	% of residents with access to exercise opportunities	% of housing units in rural areas
Median household income	1.0000										
% of residents who are uninsured	-0.3427	1.0000									
Unemployment rate	-0.4422	0.0907	1.0000								
% of residents without a high school degree	-0.5558	0.5691	0.4332	1.0000							
Food environment index	0.5811	-0.3734	-0.4135	-0.3536	1.0000						
% of residents who are non-white	-0.1626	0.2943	0.3128	0.3318	-0.5025	1.0000					
% of residents who are non-citizen	0.2396	0.3398	-0.0503	0.3161	0.1092	0.1968	1.0000				
Number of membership associations per 1,000 population	-0.0635	-0.0533	-0.2409	-0.1955	0.0857	-0.2240	-0.2042	1.0000			
% of households with severe housing problems	-0.0748	0.1649	0.2691	0.2192	-0.2488	0.4838	0.4002	-0.4056	1.0000		

© 2021 Zhang Y et al. *JAMA Network Open.*

% of residents with access to exercise opportunities	0.4109	-0.2906	-0.1420	-0.3582	0.3126	-0.0797	0.2550	-0.0751	0.2209	1.0000	
% of housing units in rural areas	-0.3897	0.1693	0.0897	0.1788	-0.1159	-0.2009	-0.4042	0.2663	-0.3595	-0.6094	1.0000

© 2021 Zhang Y et al. *JAMA Network Open.*

eTable 6. Sources of social determinants of health measures used in this study

Measures	Source	Year of Data
Median household income	American Community Survey	2017
Uninsured rate	American Community Survey	2017
Unemployment rate	American Community Survey	2017
% without high school degree	American Community Survey	2017
Food environment index[1]	2019 County Health Rankings and Roadmaps	2015 & 2016
% of Hispanic	American Community Survey	2017
% of non-Hispanic black	American Community Survey	2017
% of non-Hispanic with another race [2]	American Community Survey	2017
% of residents who are non-citizen	American Community Survey	2017
Social associations per 1,000 population[3]	2019 County Health Rankings and Roadmaps	2016
% of households with severe housing problems[4]	2019 County Health Rankings and Roadmaps	2011-2015
% of residents with access to exercise opportunities[5]	2019 County Health Rankings and Roadmaps	2010 & 2018
% of housing units in rural areas	Decennial Census	2010

Notes: [1] Food environment index equally weights two indicators of the food environment:(1) Limited access to healthy foods, which estimates the percentage of the population that is low income and does not live close to a grocery store. (2) Food insecurity, which estimates the percentage of the population that did not have access to a reliable source of food. [2]Other races include American Indian and Alaska Native, Asian, Native Hawaiian and Other Pacific Islander, and other races. [3]Social Associations measures the number of membership associations per 10,000 population. [4] Severe housing problems is the percentage of households with one or more of the following housing problems: (1) Housing unit lacks complete kitchen facilities; (2) Housing unit lacks complete plumbing facilities; (3) Household is overcrowded; or (4) Household is severely cost burdened. [5] Access to exercise opportunities measures the percentage of individuals in a county who live reasonably close to a location for physical activity, defined as parks or recreational facilities. Individuals are considered to have access to exercise opportunities if they reside in a census block that is within a half mile of a park, or reside in an urban census block that is within one mile of a recreational facility, or reside in a rural census block that is within three miles of a recreational facility. More information about these measures could be found at: https://www.countyhealthrankings.org/explore-health-rankings/measures-data-sources/2021-measures

© 2021 Zhang Y et al. *JAMA Network Open.*

eAppendix 2. Regression models to examine the contribution of patient, supply, and SDoH characteristics to geographic variation in per beneficiary Medicare total spending

We first categorized counties into quintiles based on their price-adjusted per beneficiary Medicare spending in 2017 and calculated the differences in mean price-adjusted per beneficiary Medicare spending between each higher spending quintile (quintiles 2-5) and quintile 1. We then followed previously developed methods to examine the extent to which the variation in price-adjusted per beneficiary Medicare spending across quintiles could be explained by (1) patient demographics, (2) patient clinical risk, (3) supply of health resources, and (4) SDoH.

To assess the total contribution of each group of characteristics to geographic variation in Medicare spending, we first ran a linear regression model where the outcome variable is the price-adjusted per beneficiary spending and explanatory variables are one of the four groups of characteristics above.

$$Y_i = \alpha + \beta X_i + \varepsilon_i \ (1)$$

In this equation, Y_i represents the price-adjusted per beneficiary Medicare spending in each county i, X_i is a vector of independent variables (e.g., demographics or clinical risk). β represents the coefficients estimating the relationship between per beneficiary Medicare spending and the independent variables. ε_i represents the error term. This model is weighted by the number of fee-for-service patients in each county.

After estimating model (1) using OLS, we estimated the predicted value of the outcome $\hat{Y}_i$ given the independent variables and estimated coefficients $\hat{\beta}$ and calculated the residual for each county as $e_i = Y_i - \hat{Y}_i$. e_i represents the per beneficiary spending that is not explained by independent variables. We then calculated the mean per beneficiary spending across all counties as $\bar{Y} = \sum_{i=1}^{3,038} Y_i$. Finally, the adjusted per beneficiary spending for each county was calculated as $\hat{Y}_{adj\_i} = \bar{Y} + e_i$,which removes variation in Y_i explained by X_i. The adjusted variation in per beneficiary spending was calculated as the differences in mean $\hat{Y}_{adj\_i}$ among counties in quintiles 2-5 and mean $\hat{Y}_{adj\_i}$ among counties in quintile 1. If the independent variables in the regression model (1) could explain the variation, we would expect a narrowed variation across quintiles. The share of the variation explained by the independent variable was calculated as one minus the ratio of variation in adjusted spending $\hat{Y}_{adj\_i}$ to that in price-adjusted per beneficiary spending Y_i, times 100. This model and the estimation process were repeated for four times to calculate the total contribution of each group of characteristics.

We note that this approach is analogous and yields similar results to the R-squared statistic of the regression with Y_i as the dependent variable and X_i as independent variables (Figure 3 and Table 2). The current approach has the benefit of allowing us to flexibly present changes in spending in terms of dollar amounts of counties in different spending quintiles.

To estimate the direct contribution of each group of characteristics, we ran a single model using all characteristics as independent variables.

$$Y_i = \alpha + \beta_1 demographic_i + \beta_2 clinical\ risk_{\ i} + \beta_3\ supply_i + \beta_4 SDoH_i + \varepsilon_i \ (2)$$

© 2021 Zhang Y et al. *JAMA Network Open.*

Similar with model 1, we first calculated the residual for each county as $e_i = Y_i - \hat{Y}_i$ after estimating model (1) using OLS. We then sequentially replaced each group of characteristics using their means across all counties and estimated the predicted per beneficiary spending $\hat{Y}_i$ given the independent variables and estimated coefficients $\widehat{\beta_1} - \widehat{\beta_4}$. Finally, the adjusted per beneficiary spending is calculated as $\hat{Y}_{adj_i} = \hat{Y}_i + e_i$. Similarly, the adjusted variation in per beneficiary spending was calculated as the differences in mean $\hat{Y}_{adj\_i}$ among counties in quintiles 2-5 and mean $\hat{Y}_{adj\_i}$ among counties in quintile 1. The share of the variation explained by the independent variable was calculated as one minus the ratio of variation in adjusted spending $\hat{Y}_{adj\_i}$ to that in price-adjusted per beneficiary spending Y_i, time 100. This process was repeated four times to calculate the direct contribution of each group of characteristics.

eTable 7. Summary of regression models and their purposes

Model	Outcome	Independent Variables	Purposes	Primary or Sensitivity
1	Price-adjusted per beneficiary spending	Demographics	Estimating total contribution of demographics	Primary
2		Clinical risk	Estimating total contribution of clinical risk	Primary
3		Supply	Estimating total contribution of supply of healthcare	Primary
4		SDoH	Estimating total contribution of SDoH	Primary
5		Demographics, clinical risk, supply, and SDoH	Estimating direct contribution of each set of characteristics	Primary
6	Price-adjusted per beneficiary spending	Demographics, supply, and SDoH	Estimating direct contribution of demographics, supply, and SDoH	Sensitivity
7	Price-, age-, gender-, and race-adjusted per beneficiary spending	Supply and SDoH	Estimating direct contribution of supply and SDoH	Sensitivity

© 2021 Zhang Y et al. *JAMA Network Open.*

eFigure 1. Contribution to variation in price-adjusted per beneficiary Medicare spending between quintiles 2-5 and quintile 1, excluding clinical risk score

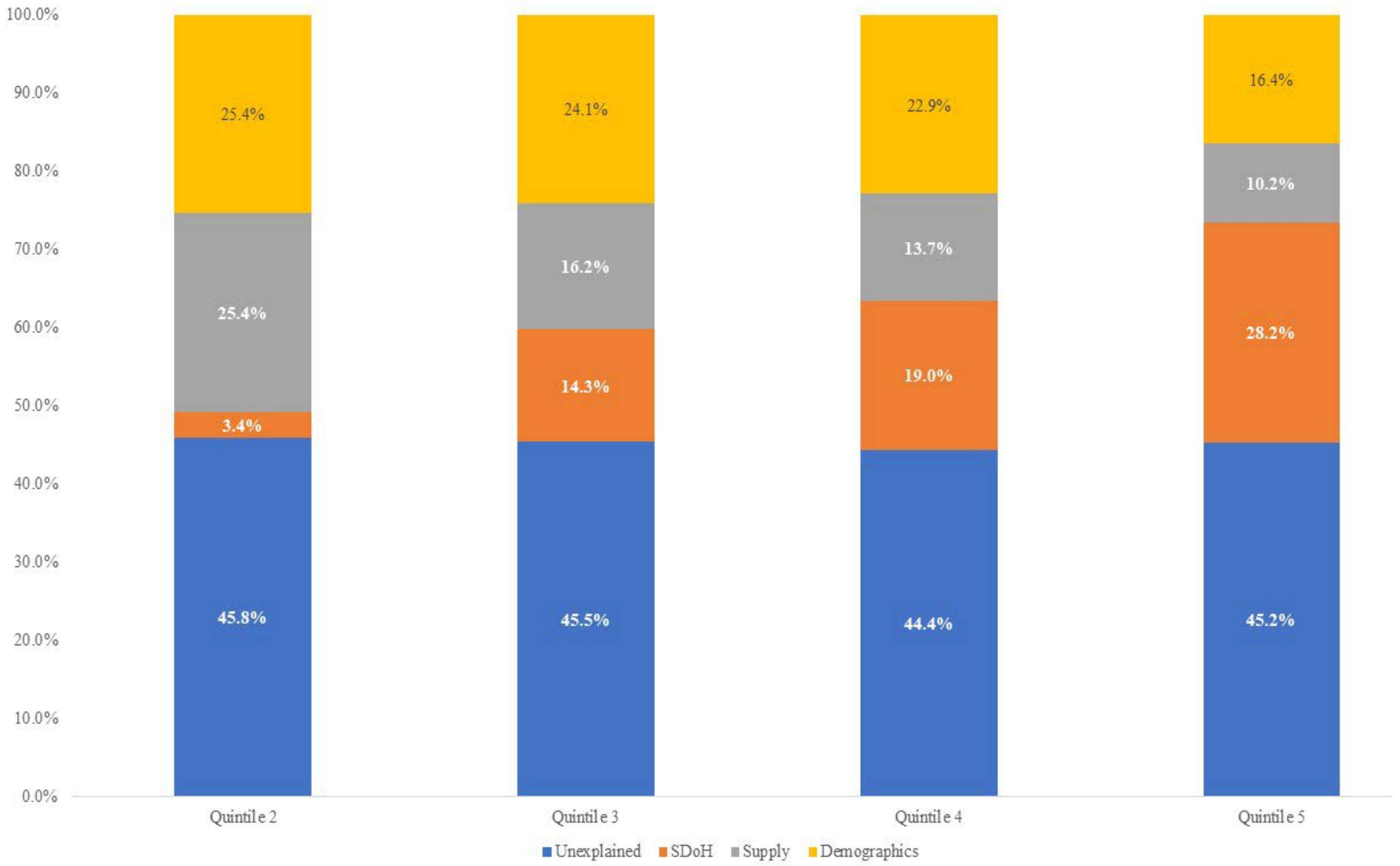

Notes: For each quintile, the share of variation associated with each set of characteristics was estimated when controlling for other characteristics. Demographics include age, age squared, age cubed, and gender; supply characteristics include the following measures per 1,000 population: primary care physicians, specialists, hospital beds, skilled nursing facility beds, home health agency aides, registered nurses employed by hospices, and ambulatory care centers. SDoH include median household income, % who are uninsured, unemployment rate, % without high school degree, food environment index; % who are Hispanic, % of non-Hispanic black, and % of non-Hispanic with another race (i.e., American Indian and Alaska Native, Asian, Native Hawaiian and Other Pacific Islander, and other races), % who are non-citizen, social associations per 1,000 population, % with severe housing problems, % with access to exercise opportunities, and % of housing units in rural areas.

© 2021 Zhang Y et al. *JAMA Network Open.*

eFigure 2. Contribution to variation in price-, age-, gender-, and race-adjusted per beneficiary spending between quintiles 2-5 and quintile 1, using Dartmouth spending as outcome

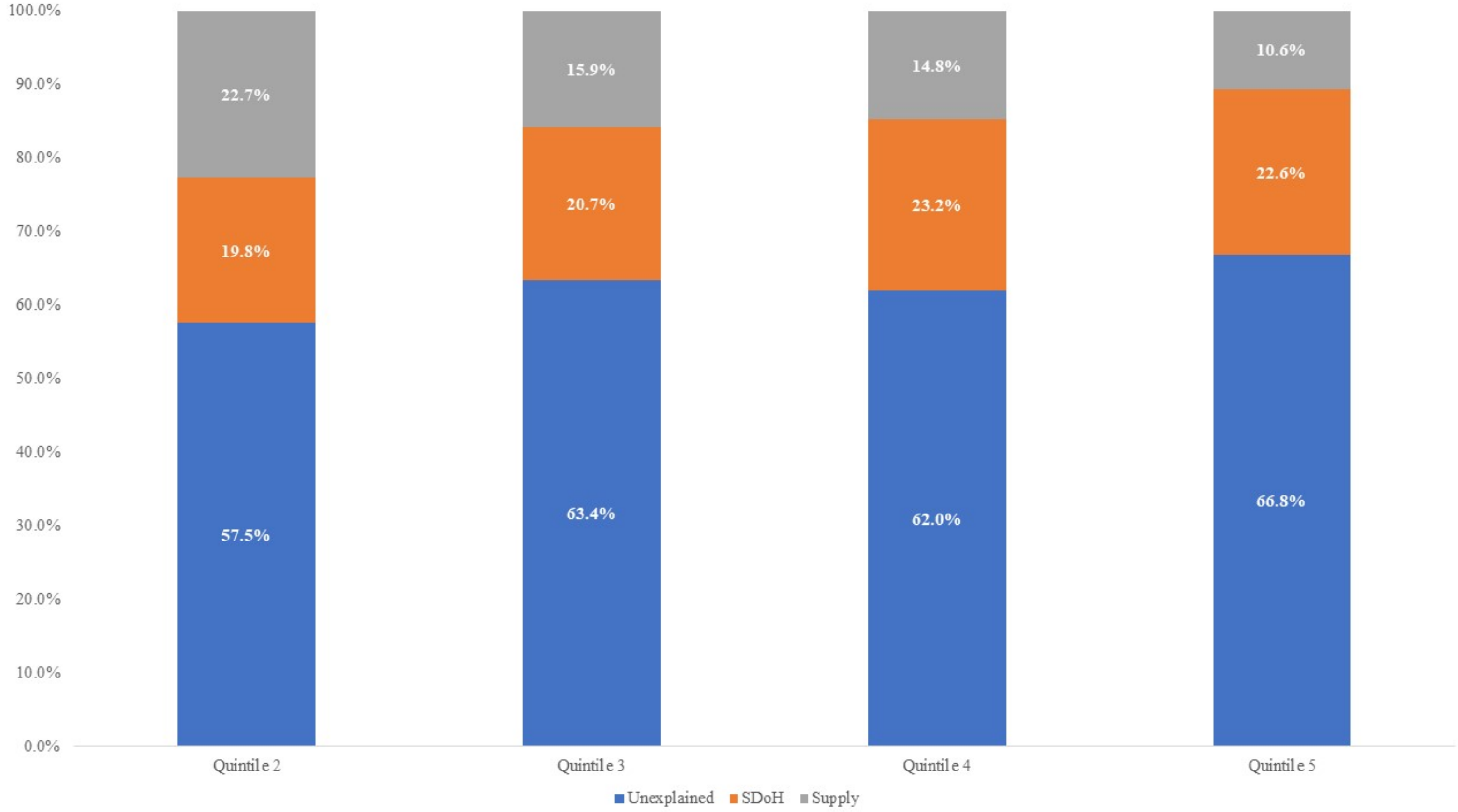

Notes: For each quintile, the share of variation associated with each set of characteristics was estimated when controlling for other characteristics. Supply characteristics include the following measures per 1,000 population: primary care physicians, specialists, hospital beds, skilled nursing facility beds, home health agency aides, registered nurses employed by hospices, and ambulatory care centers. SDoH include median household income, % who are uninsured, unemployment rate, % without high school degree, food environment index; % who are Hispanic, % of non-Hispanic black, and % of non-Hispanic with another race (i.e., American Indian and Alaska Native, Asian, Native Hawaiian and Other Pacific Islander, and other races), % who are non-citizen, social associations per 1,000 population, % with severe housing problems, % with access to exercise opportunities, and % of housing units in rural areas.

© 2021 Zhang Y et al. *JAMA Network Open.*

eTable 8. Full regression output of sensitivity analysis: coefficients and robust standard errors

	Using CMS spending measure	Partial F (p value)	Using Dartmouth spending measure	Partial F (p value)
Demographics				
Age	-103884.8 * (51690.18)	48.6 (<0.001)	–	–
Age squared	1440.14 (734.07)			
Age cubed	-6.65 (3.47)			
% of female	262.87 *** (24.08)		–	
Supply of health resources per 1,000 population Mean				
Number of PCPs	-1122.92 *** (160.14)	28.7 (<0.001)	-1018.85 *** (165.76)	30.2 (<0.001)
Number of specialists	93.56 ** (34.92)		74.86 * (31.47)	
Number of hospital beds	37.25 *** (9.77)		38.35 *** (10.96)	
Number of SNF beds	89.29 *** (8.01)		104.14 *** (8.44)	
Number of HHA aides	43.46 (28.42)		34.58 (24.87)	
Number of hospice RNs	329.85 (174.74)		419.48 * (196.50)	
Number of ASCs	-6738.00 *** (1896.45)		-7535.31 *** (2096.51)	
Social determinants of health				
Socioeconomic Position				
Median household income ($)	-0.001 (0.005)	53.0 (<0.001)	0.002 (0.006)	43.1 (P<0.001)
Uninsured rate	103.56 *** (13.25)		95.47 *** (14.29)	
Unemployment rate	74.39 * (37.64)		41.30 (41.46)	
% without high school degree	20.26 (18.59)		49.45 * (21.03)	
Food environment index	135.88 (69.71)		261.83 ** (89.04)	
Race & Ethnicity				
% of residents who are non-citizens	-30.49 (20.74)		-17.44 (23.46)	
% of residents who are Hispanic	1.84 (4.91)		-14.34 ** (5.32)	
% of residents who are non-Hispanic black	15.96 *** (3.69)		9.65 * (4.82)	
% of residents who are non-Hispanic other races	-43.27		-35.43	

© 2021 Zhang Y et al. *JAMA Network Open.*

	(169.18)		(188.42)	
Social Relationships				
Social associations per 1,000 population	-55.20 *** (9.59)		-70.77 *** (10.47)	
Residential and Community Context				
% of households with severe housing problems	18.78 (22.48)		3.06 (25.60)	
% of residents with access to exercise opportunities	-4.12 (2.24)		-5.57 (2.50) *	
% of housing units in rural areas	-11.81 *** (2.02)		-17.26 *** (1.89)	
N	3,038		3,038	
Overall F	67.7		38.8	
p-value of F	<0.001		<0.001	
R-squared	0.53		0.33	
Adjusted R-squared	0.54		0.33	

Notes: PCP: primary care physicians; SNF: skilled nursing facility; HHA: home health agency; RN: registered nurses; ASC: ambulatory surgery center. * P<0.05, **P<0.01, ***P<0.001. Results are from the linear regressions using CMS price-adjusted per beneficiary Medicare spending or Dartmouth price-, age-, gender-, and race-adjusted spending as outcome, controlling for variables in each column in the regression models. We reported coefficients and robust standard errors for each variable.

© 2021 Zhang Y et al. *JAMA Network Open.*

eTable 9. Full regression output of the association of CMS-HCC score with SDoH, demographics, and supply of healthcare sources: coefficients and robust standard errors

	SDoH	F tests	SDoH and demographics	Partial F tests	SDoH, demographics, and supply of health resources	Partial F tests
Social determinants of health						
Socioeconomic Position						
Median household income ($1,000)	-0.002 *** (0.0005)	68.8 (P<0.001)	-0.002 *** (0.0004)	38.40 (P<0.001)	-0.001 ** (0.0004)	36.51 (P<0.001)
Uninsured rate	-0.0009 (0.0009)		0.0001 (0.0009)		0.001 (0.001)	
Unemployment rate	0.002 (0.003)		0.007 ** (0.003)		0.007 ** (0.003)	
% without high school degree	0.008 *** (0.001)		0.005 *** (0.001)		0.004 ** (0.001)	
Food environment index	0.024 *** (0.006)		0.016 ** (0.005)		0.016 ** (0.006)	
Race & Ethnicity						
% of residents who are non-citizens	-0.0005 (0.002)		-0.001 (0.001)		-0.0003 (0.001)	
% of residents who are Hispanic	-0.0008 (0.0004)		0.0001 (0.0004)		0.0001 (0.0004)	
% of residents who are non-Hispanic black	0.002 *** (0.0003)		0.001 *** (0.0003)		0.001 *** (0.0003)	
% of residents who are non-Hispanic with another races	0.018 (0.010)		0.013 (0.009)		0.001 (0.010)	
Social Relationships						
Social associations per 1,000 population	-0.0005 (0.0006)		-0.002 * (0.0007)		-0.003 *** (0.0007)	
Residential and Community Context						
% of households with severe housing problems	-0.003 * (0.001)		0.004 ** (0.001)		0.005 *** (0.001)	
% of residents with access to exercise opportunities	-0.0002 (0.0002)		-0.0002 (0.0002)		-0.0001 (0.0002)	

© 2021 Zhang Y et al. *JAMA Network Open.*

% of housing units in rural areas	-0.002 *** (0.0001)		-0.0008 *** (0.0001)		-0.001 *** (0.0001)	
Demographics						
Age			-3.31 (3.44)	35.65 (P<0.001)	-2.53 (3.15)	29.71 (P<0.001)
Age squared			0.001 *** (0.0003)		0.03 (0.04)	
Age cubic					-0.0001 (0.0002)	
% of female			0.016 *** (0.0009)		0.018 *** (0.002)	
Supply of health resources per 1,000 population Mean						
Number of PCPs					-0.058 *** (0.012)	19.79 (P<0.001)
Number of specialists					0.006 * (0.003)	
Number of hospital beds					0.002 ** (0.0006)	
Number of SNF beds					0.005 *** (0.0006)	
Number of HHA aides					0.003 (0.002)	
Number of hospice RNs					-0.007 (0.013)	
Number of ASCs					-0.589 *** (0.140)	
N	3,038		3,038		3,038	
R-squared	0.55		0.63		0.65	
Adjusted R-squared	0.54		0.61		0.64	

Notes: PCP: primary care physicians; SNF: skilled nursing facility; HHA: home health agency; RN: registered nurses; ASC: ambulatory surgery center. * P<0.05, **P<0.01, ***P<0.001. Results are from the linear regressions using CMS-HCC scores as the outcome. We reported coefficients and robust standard errors for each variable.

© 2021 Zhang Y et al. *JAMA Network Open.*

eFigure 3. Contribution to variation in CMS HCC score between quintiles 2-5 and quintile 1

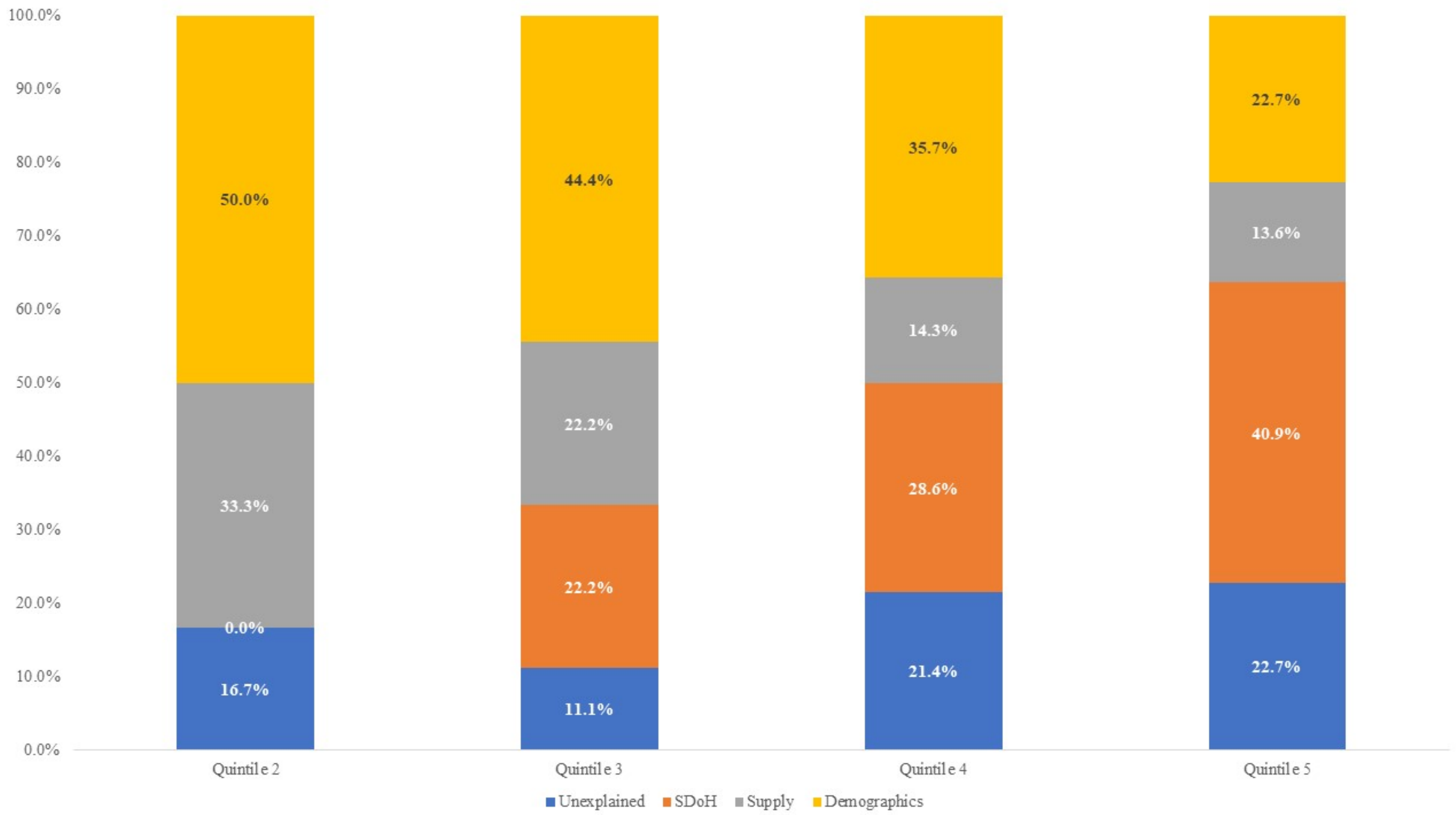

Notes: For each quintile, the share of variation associated with each set of characteristics was estimated when controlling for other characteristics. Demographics include age, age squared, age cubed, and gender. Supply characteristics include the following measures per 1,000 population: primary care physicians, specialists, hospital beds, skilled nursing facility beds, home health agency aides, registered nurses employed by hospices, and ambulatory care centers. SDoH include median household income, % who are uninsured, unemployment rate, % without high school degree, food environment index; % who are Hispanic, % of non-Hispanic black, and % of non-Hispanic with another race (i.e., American Indian and Alaska Native, Asian, Native Hawaiian and Other Pacific Islander, and other races), % who are non-citizen, social associations per 1,000 population, % with severe housing problems, % with access to exercise opportunities, and % of housing units in rural areas.

References:

1. National Academies of Sciences E, Medicine. *Accounting for social risk factors in Medicare payment.* National Academies Press; 2017.
2. Kibria A, Mancher M, McCoy MA, Graham RP, Garber AM, Newhouse JP. *Variation in health care spending: target decision making, not geography.* National Academies Press; 2013.
3. Baker LC, Bundorf MK, Kessler DP. Patients' preferences explain a small but significant share of regional variation in medicare spending. *Health Aff (Millwood).* 2014;33(6):957-963.

© 2021 Zhang Y et al. *JAMA Network Open.*

4. Finkelstein A, Gentzkow M, Williams H. Sources of Geographic Variation in Health Care: Evidence from Patient Migration. *Q J Econ.* 2016;131(4):1681-1726.
5. Reschovsky JD, Hadley J, Saiontz-Martinez CB, Boukus ER. Following the money: factors associated with the cost of treating high-cost Medicare beneficiaries. *Health Serv Res.* 2011;46(4):997-1021.
6. Patrick SW, Choi H, Davis MM. Increase in federal match associated with significant gains in coverage for children through Medicaid and CHIP. *Health Aff (Millwood).* 2012;31(8):1796-1802.

© 2021 Zhang Y et al. *JAMA Network Open.*
